# Genome-wide conserved consensus transcription factor binding motifs are hyper-methylated

**DOI:** 10.1186/1471-2164-11-519

**Published:** 2010-09-27

**Authors:** Mun-Kit Choy, Mehregan Movassagh, Hock-Guan Goh, Martin R Bennett, Thomas A Down, Roger SY Foo

**Affiliations:** 1 Department of Medicine, University of Cambridge, ACCI Building Level 6, Cambridge, CB2 0QQ, UK; 2 Department of Computer and Communication Technology, Faculty of Information, Communication and Technology, University of Tunku Abdul Rahman, Perak, Malaysia; 3 The Gurdon Institute and Department of Genetics, University of Cambridge, Cambridge CB2 1QN, UK

## Abstract

**Background:**

DNA methylation can regulate gene expression by modulating the interaction between DNA and proteins or protein complexes. Conserved consensus motifs exist across the human genome ("predicted transcription factor binding sites": "predicted TFBS") but the large majority of these are proven by chromatin immunoprecipitation and high throughput sequencing (ChIP-seq) not to be biological transcription factor binding sites ("empirical TFBS"). We hypothesize that DNA methylation at conserved consensus motifs prevents promiscuous or disorderly transcription factor binding.

**Results:**

Using genome-wide methylation maps of the human heart and sperm, we found that all conserved consensus motifs as well as the subset of those that reside outside CpG islands have an aggregate profile of hyper-methylation. In contrast, empirical TFBS with conserved consensus motifs have a profile of hypo-methylation. 40% of empirical TFBS with conserved consensus motifs resided in CpG islands whereas only 7% of all conserved consensus motifs were in CpG islands. Finally we further identified a minority subset of TF whose profiles are either hypo-methylated or neutral at their respective conserved consensus motifs implicating that these TF may be responsible for establishing or maintaining an un-methylated DNA state, or whose binding is not regulated by DNA methylation.

**Conclusions:**

Our analysis supports the hypothesis that at least for a subset of TF, empirical binding to conserved consensus motifs genome-wide may be controlled by DNA methylation.

## Background

DNA methylation is a well-studied component of epigenetics that, in the mammalian system, involves the 5' covalent modification of cytosine nucleotides by a methyl group. In humans, cytosine methylation almost always occurs in the context of a CG di-nucleotide, except in undifferentiated cells where methylation was recently identified in cytosines that do not precede guanines (non-CG methylation) [1,2]. Regions of high CG density, termed "CpG islands" are usually un-methylated and found mainly in the 5' promoter ends of genes. However high resolution maps of genome-wide methylation now show that cytosine methylation occurs throughout the genome, particularly in bodies of highly expressed genes [3], and up to 4.25% of cytosines in the human genome are methylated [1]. Although the functional difference between CG and non-CG methylation requires further investigation, it is clear that DNA methylation itself significantly regulates gene expression and affects cellular processes in disease and development [4]. For example, genome-wide methylation is altered during aging [5-7] and malignant transformation [8], and recent evidence supports the notion that methylation can be modulated by diet and environment [9-11]. Moreover evidence of rapid and dynamic DNA methylation/de-methylation *in vivo *[12,13] challenges the conventional view that DNA methylation is a stable or permanent epigenetic mark.

Mechanisms to explain aberrant *de novo *methylation in these contexts include (a) targeted recruitment of DNA methyl-transferases by *cis*-acting factors such as G9a or EZH2 [14], or (b) loss of boundaries or "protective" transcription factors leading to the spread of DNA methylation into affected regions in the genome [15,16]. Indeed several non-redundant sequences matching the consensus motifs for transcription factors such as SP1 have been identified at sites that are resistant to *de novo *methylation in cancer [17]. *De novo *methylated CpG islands in cancer however were characterized by the lack of sequence motif combinations and the absence of activating TF binding [17].

Conversely, the classical mechanism by which DNA methylation regulates transcription is through altered accessibility of transcription factor complexes to their cognate DNA binding sites [4,18]. This mechanism is supported by many locus-specific examples [19,20] but one that links the mechanism to environmental influences is the rodent model of maternal grooming [10]. "Highly groomed" neonates developed hypo-methylation in the first exon of the glucocorticoid receptor gene which in turn permits binding of the transcription factor NGFI-A to this DNA regulatory region and up-regulates glucocorticoid receptor expression [10]. In contrast, "lesser groomed" neonates developed methylation in the same DNA regulatory sequence with corresponding inhibition of NGFI-A binding and down-regulation of glucocorticoid receptor expression.

Conserved consensus motifs have been predicted for transcription factor binding across the human genome, and empirical transcription factor binding sites (TFBS) have been determined biologically using the genome-wide technique which couples chromatin immunoprecipitation and high throughput sequencing (ChIP-seq). We have previously examined the genome-wide methylome of human hearts [21] and sperm [22]. We therefore set out to analyze the methylation state of TFBS in these methylation maps.

## Results

### Conserved transcription factor consensus motifs (predicted TFBS) are hyper-methylated

We analyzed genome-wide DNA methylation profile in 4 normal adult human hearts (a post-mitotic organ) and human sperm (germ cell) by employing the technique of MeDIP-seq [22]. Analysis of our MeDIP-seq datasets was performed using the Bayesian deconvolution algorithm called BATMAN [22]. Using BATMAN we assigned methylation scores across the genome for hearts and sperm. Because we hypothesized that the interaction between transcription factor complexes and their cognate DNA binding sites is modulated and influenced by methylation of cytosines within the DNA sequence [4,18], we examined methylation profiles at genome-wide sites of transcription factor binding.

**Figure 1:**
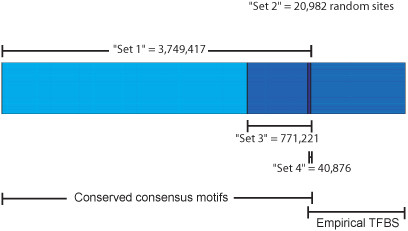
**Datasets of genomic locations**. Set 1: Predicted TFBS based on conserved consensus motifs for 106 transcription factor families (N = 3,749,417). Set 2: Random genomic locations (N = 20,982). Set 3: Predicted TFBS for 17 transcription factor families (N = 771,221). Set 4: Biologically proven TFBS (empirical TFBS by ChIP-seq) with conserved consensus motifs (N = 40,876).

**Figure 2:**
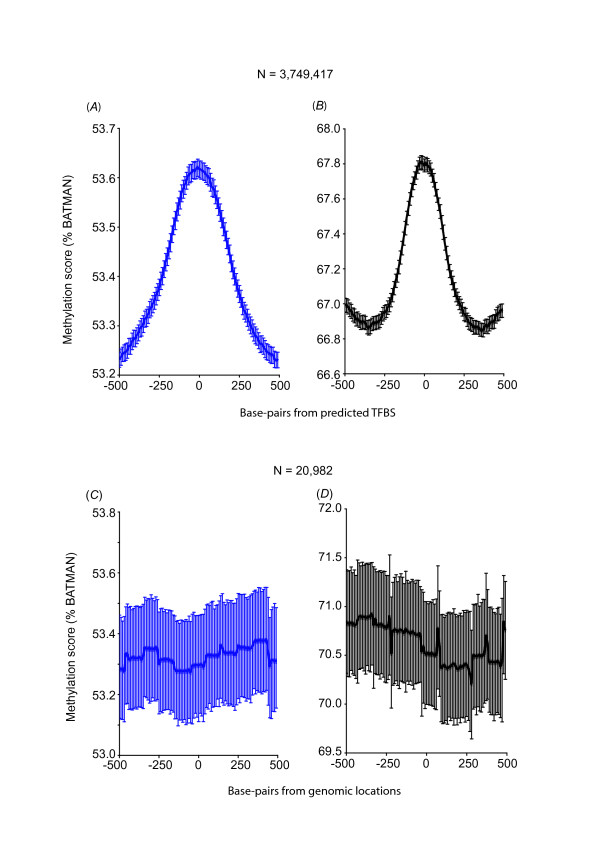
**Genome-wide conserved consensus motifs (predicted TFBS) are hyper-methylated in human hearts and sperm**. Methylation scores were determined across the genome for hearts and sperm using BATMAN [21]. The profiles of these scores (*A*: hearts, *B*: sperm) were plotted against locations of conserved consensus motifs for 106 transcription factor families centred on the predicted TF binding site (based on co-ordinates obtained from the UCSC genome web browser: TFBS Conserved track; "set 1", N = 3,749,417 locations). Methylation profile at random genomic locations was analyzed as a negative control and reflects a "neutral" methylation pattern at these locations ("set 2", N = 20,982).

First, we made use of a computational dataset of transcription factor motifs where locations of motifs were determined based on a score which met the threshold for its conserved binding matrix in the alignment for all 3 species: human, rat and mouse (HMR) http://www.gene-regulation.com. Score and threshold were computed with the Transfac Matrix Database (v7.0), created by Biobase, and are currently found on the UCSC genome web browser (HMR Conserved TFBS, we called "set 1", Figure 1). We observed a profile of increased average methylation, centred on the predicted TFBS at 3,749,417 locations where the consensus motifs for 106 transcription factor families were conserved (Figure 2A, Table 1 for list of 106 transcription factor families). A similar methylation profile was found for the same 3,749,417 genomic locations in sperm cells (Figure 2B). In contrast, a separate control analysis performed with a set of random genomic locations (N = 20,982, we called "set 2", Figure 1) showed no modulation in the methylation profile across random genomic locations in both hearts and sperm (Figures 2C and 2D). When examined in further detail for each individual TF, the large majority of TF showed a hyper-methylation profile in both hearts and sperm (Table 1) but a smaller proportion showed a variation of hypo-methylation pattern in both tissues, an opposite pattern in either, or a neutral methylation pattern. Although the latter analysis implicates the possibility that binding of sub-groups of transcription factors are variably affected by DNA methylation pattern, and differences between hearts and sperm may exist for specific sets of TF, the aggregate hyper-methylation profile in the former analysis suggests that conserved consensus TF motifs are mainly hyper-methylated.

**Table 1:** List of 106 transcription factor families (from UCSC genome web browser, Conserved TFBS track) and their detailed methylation profiles in hearts and sperm.

		SPERM	
		Hyper-methylated	Hypo-methylated
**HEART**	**Hyper-methylated**	AHR-ARNT, AML1, AP, AREB6, ARP1, ATF, BACH, BRACH, CDP, CEBP, CHOP, COMP1, COUP, CP2, CREB, EN1, ER, FAC1, GATA, GCNF, GFI1, GR, HEN1, HMX1, HOX, HSF, HTF, IK, ISRE, LMO2COM, LUN1, LYF1, MEIS1, MIF1, MRF2, MSX1, MYB, MYCMAX, MYOD, MYOGNF1, MZF1, NCX, NF1, NFE2, NFKB, NRSF, OLF1, EP300, P53, PAX, PBX1, PPAR, RFX1, ROAZ, RORA, RP58, RREB1, SEF1, SPZ1, SREBP, SRF, STAT, TAL1-E47, TCF, TGIF, USF, XBP1, YY1, ZIC, ZID	-
	
	**Hypo-methylated**	CETS1P54, E2F, E4BP4, EGR, ELK1, MAZR, NFY, NRF1, SRY	CHX10, FOX, FREAC, LHX3, MEF2, POU, RSRFC4, S8, HFH, SOX, SP1, TATA, TBP
	
	**Neutral**	BRN2, CREL, HLF, IRF, NFAT, NGFIC, NMYC, TST1 CDC5, OCT	CART1, NKX, EVI1, HNF

### Empirical TFBS with conserved consensus motifs are hypo-methylated

**Figure 3:**
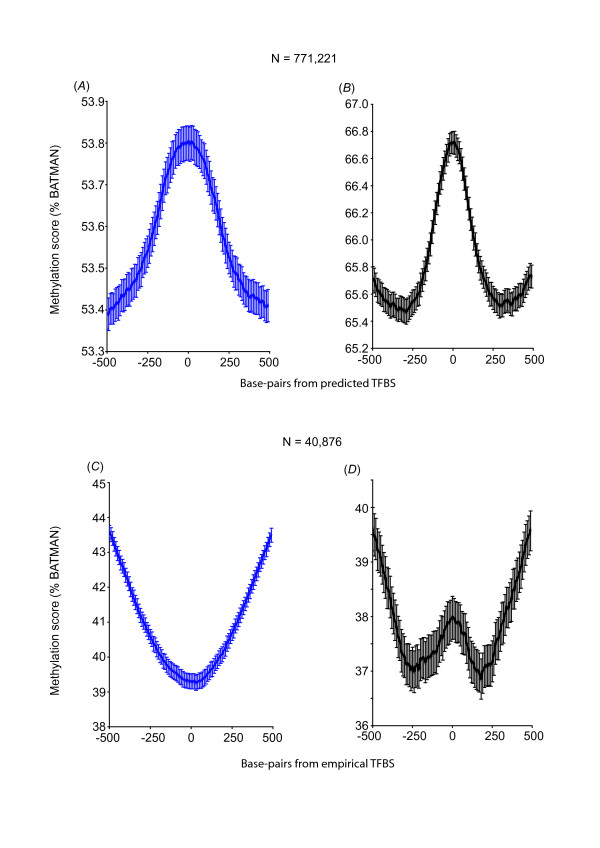
**Predicted TFBS with conserved consensus motifs are hyper-methylated unless they are also biologically proven TFBS (empirical TFBS)**. Aggregate methylation profiles for predicted TFBS with conserved consensus motifs for a subset of 17 transcription factor families are hyper-methylated in hearts (*A*) and sperm (*B*) (N = 771,221); but hypo-methylated if they are also biologically proven TFBS (empirical TFBS by ChIP-seq; "set 4", N = 40,876) (*C*: hearts and *D*: sperm).

Empirical or *bona fide *genome-wide sites of transcription factor binding are now determined by ChIP-seq [23] and the ENCODE consortium http://www.genome.gov/10005107 has now performed ChIP-seq for at least 17 different TF. We therefore examined predicted TFBS (HMR Conserved TFBS) for these 17 TF (see Table 2 for the list of these 17 TF). At the locations of predicted TFBS for this subset of 17 TF (N = 771,221, we called "set 3", Figure 1), a hyper-methylation profile (Figures 3A and 3B) was again found in both hearts and sperm.

**Table 2:** List of 17 transcription factor families from ENCODE (UCSC genome web browser) and other published sources, and their detailed methylation profiles in hearts and sperm.

Motif family	Heart	Sperm
E2F^1^	Hypo-methylated	Hypo-methylated
NFY^1^	Hypo-methylated	Hypo-methylated
YY1^1^	Hypo-methylated	Hypo-methylated
MYCMAX^1^	Hypo-methylated	Hyper-methylated
NFKB^1^	Hypo-methylated	Hyper-methylated
AP^1^	Hypo-methylated	Hyper-methylated
NRSF^2^	Hypo-methylated	Neutral
SREBP^3^	Hypo-methylated	Neutral
SRF^2^	Hypo-methylated	Neutral
STAT^4^	Hypo-methylated	Neutral
TCF^1^	Hypo-methylated	Neutral
GATA^1^	Neutral	Hyper-methylated
NFE2^1^	Neutral	Neutral
OCT^5^	Neutral	Neutral
SOX^5^	Neutral	Neutral
EP300^5^	Neutral	Neutral
TP53^6^	Neutral	Neutral

^1^Yale/UCD/Harvard (ENCODE)

^2^HudsonAlpha Institute

^3^Yale/UCD/Harvard/Duke UNC/UT, Genome Institute of Singapore (GIS)

^4^Yale/UCD/Harvard, GIS

^5^Lister et al, Nature 2009

^6^GIS

Next we compared predicted TFBS (HMR Conserved TFBS) to empirical TFBS (ENCODE ChIP-seq) for the same 17 TF. This revealed that only 40,876 locations were both predicted TFBS and empirical TFBS (empirical TFBS containing the expected conserved consensus motif, we called "set 4", Figure 1); i.e. 3.4% (40,876 out of 1,187,431) of the empirical TFBS were predicted by motif and conservation, and 5.3% (40,876 out of 771,221) of the predicted TFBS were biologically proven TBFS as determined by ChIP-seq. In contrast to the aggregate hyper-methylation profile at all predicted TFBS ("set 1", Figures 2A and 2B) and at the subset of 17 TFBS ("set 3", Figures 3A and 3B), predicted TFBS that were biologically proven TFBS ("set 4") showed an aggregate profile of hypo-methylation in both hearts and sperm (Figures 3C and 3D). Table 2 shows the detailed methylation profile for each TF in "set 4". All TF were associated with either a hypo-methylation or neutral profile in hearts whereas 3 out of 17 TF showed a hyper-methylation profile in sperm. The latter detailed analysis may reflect specific differences in empirical TF binding between a post-mitotic organ and germ cell.

### Empirical TFBS with conserved consensus motif are more likely to reside in CpG islands than predicted TFBS

CpG islands (CGI) are CG-rich genomic regions often located at the 5' promoter region of genes. Since CpG islands are largely hypo-methylated [1,2] and the interaction between transcription factor complexes and DNA may be regulated by CGI/promoter methylation, we asked what proportion of our sets of genomic locations corresponded to CGI. Only 7% of the subset of 17 predicted TFBS (i.e. 7% of "set 3") resided in CGI, whereas 40% of locations of empirical TFBS containing the expected conserved consensus motif (i.e. 40% of "set 4") were in CGI.

**Figure 4:**
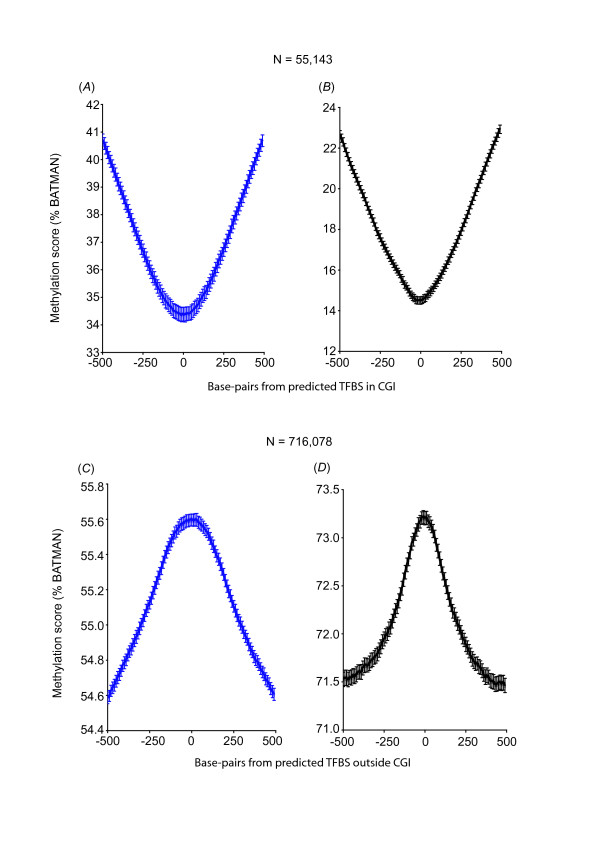
**Predicted TFBS with conserved consensus motifs residing outside of CGI are hyper-methylated**. Aggregate methylation profiles for predicted TFBS with conserved consensus motifs ("set 3") that reside in CGI (*A*: hearts, *B*: sperm), and outside of CGI (*C*: hearts, *D*: sperm) showing that conserved consensus motifs are hypo-methylated when within CGI but hyper-methylated when outside of CGI.

**Figure 5:**
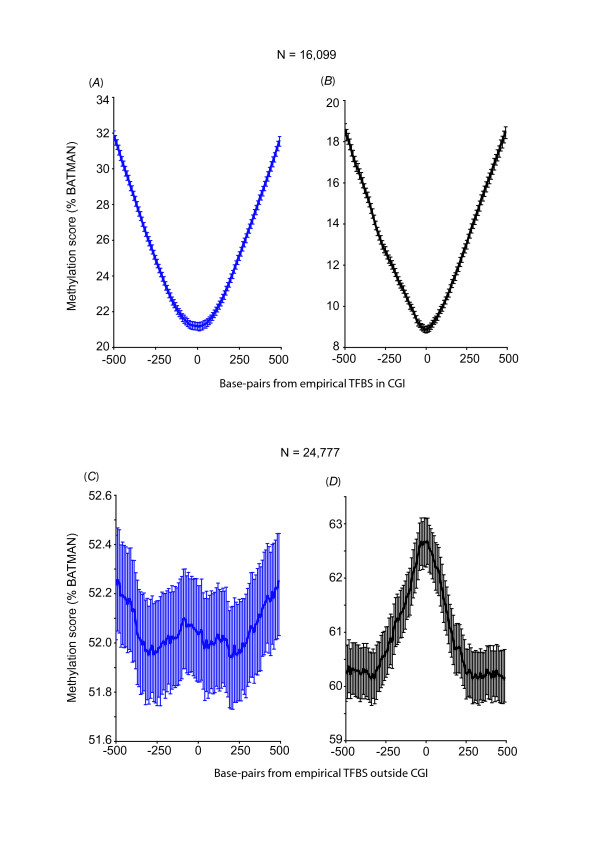
**Empirical TFBS with conserved consensus motifs are hypo-methylated in CGI but have a neutral methylation pattern when outside of CGI in the heart**. Methylation profiles for empirical TFBS with conserved consensus motifs ("set 4") that resided in CGI (*A*: hearts, *B*: sperm), and outside CGI (*C*: hearts, *D*: sperm).

We therefore divided the predicted TFBS ("set 3") into CGI and non-CGI, and examined the methylation profile for each subset. As expected, predicted TFBS in CGI were hypo-methylated (Figures 4A and 4B), whereas predicted TFBS outside of CGIs were hyper-methylated (Figures 4C and 4D). This hyper-methylation profile again suggests that where conserved consensus motifs for TF binding exist outside of CGI, promiscuous or disorderly TF binding may be controlled by DNA hyper-methylation. Similarly, although empirical TFBS containing the expected conserved consensus motif in CGI ("set 4") were hypo-methylated (Figures 5A and 5B), non-CGI of this dataset showed a hyper-methylation profile in sperm (Figure 5D) and a neutral methylation profile in hearts (Figure 5C).

## Discussion

Interaction between DNA and proteins or protein complexes can be modulated by DNA methylation. Indeed there are examples of DNA methylation-dependent binding for transcription factors such as CTCF [20] and NGFI-A [10,24]. Promiscuous or disorderly transcription factor binding may therefore be controlled by DNA methylation at potential binding sites throughout the genome where there are conserved consensus motifs. Here our global analysis largely supports this hypothesis for TF in general. Although the possibility remains that we are only sampling methylation profiles at these sites of conserved consensus motifs at a single time point, and previous or subsequent TF binding may occur as a result of dynamic changes in DNA methylation, this is the first genome-wide study to associate conserved consensus motifs (predicted TFBS) with DNA hyper-methylation. We found similar aggregate methylation profiles for the various sets of TFBS in parallel analyses using methylation maps from both hearts and sperm.

Analysis of all conserved consensus motifs throughout the genome and the subset of those that reside outside CGI showed an aggregate profile of hyper-methylation, but detailed analysis of individual TF suggests that there may be subsets of TF that behave differently. These may indeed represent specific TF whose combinatorial function is to establish or maintain the un-methylated DNA state [15,16]. Indeed our finding of SP1 and NRF1 in this latter group corresponds to previous reports [16,17,25,26] proposing this function for these two TF.

We have further found that only a very small number of predicted TFBS containing conserved consensus motif are biologically proven TBFS (empirical TFBS). Conversely, only a very small subset of empirical TFBS has the expected conserved consensus motif. Most importantly, we found that while conserved consensus motifs without biologically proven TF binding have a hyper-methylated profile, sites of biologically proven TFBS have the opposite hypo-methylation profile. Although the scales of methylation scores (% BATMAN) in our analysis are generally narrow (e.g. Figure 2, from trough to peak: 53.2 - 53.6%), these scores represent composite/aggregate scores at over 3 M locations in the genome and confidence intervals as indicated on the graphs do not show overlap from peaks to troughs, reflecting the significance of altered methylation patterns in these regions. Moreover these methylation scores are not representative of "whole-genome" methylation but only of the local regions that are being analyzed in each graph (e.g. 3, 749,417 regions in Figure 2 but a different set of 40,876 regions in Figure 3C), peak-to-trough scores therefore differ between analyses.

Interestingly, we also found that only a very small proportion of the sites of conserved consensus motif without biologically proven TF binding were within CGI s(7%); whereas a larger proportion of sites of biologically proven TF binding were within CGIs (40%). The lack of methylation modulation in sites of biologically proven TF binding outside of CGIs (Figure 5C) serves as a negative control for the other profiles of methylation differences that we have detected, but may also indicate that at these sites of empirical TF binding, a neutral methylation profile allows potential TF binding. Alternatively, potential TF binding at these sites may not be regulated by DNA methylation. Most importantly and in contrast to that, predicted consensus TFBS that are non-CGIs maintain a significant hyper-methylation pattern (Figures 4C and 4D).

## Conclusions

Our data provides genome-wide evidence that the majority of conserved consensus motifs in the human genome are hyper-methylated, whereas biologically proven TFBS with conserved consensus motifs are hypo-methylated. This implicates a role for DNA methylation in preventing promiscuous or disorderly TF binding, at least for the majority of TF.

## Methods

### Ethics Statement

Human myocardium was collected by a protocol approved by Cambridgeshire Research Ethics Committee (UK) (REC reference: 06/Q0104/64).

### Human left ventricular myocardium

Left ventricular (LV) tissue was obtained from non-donor suitable healthy male individuals involved in road traffic accidents. At the time of donor harvest, whole hearts were removed and transported in cold cardioplegic solution (cardioplegia formula and Hartmann's solution) similar to the procedure described before at Imperial College, London [27]. Following analysis by a cardiovascular pathologist, left ventricular segments were cut and immediately snap frozen.

### Genomic DNA isolation

Genomic DNA was isolated from LV samples using the Genomic DNA Buffer Set and Anionic columns (Qiagen, Crawley, UK). Samples (200 mg) were homogenized in G2 Lysis Buffer containing 80 μg/ml RNaseA, using a hand-held homogenizer (Polytron, Switzerland), and thereafter digested with 1 mg/ml Proteinase K (Roche Diagnostics, Burgess Hill, UK) overnight. Fully digested samples were centrifuged at 5000 μg for 10 min and gDNA was isolated from the supernatant using Genomic tip-500/G anionic columns (Qiagen) according to manufacturer's instructions. Integrity and purity of genomic DNA (gDNA) from each tissue was verified by Nanodrop (Thermo Scientific, Wilmington, DE) and the QIAxcel system (Qiagen).

### Methylated-cytosine DNA Immunoprecipitation - high throughput sequencing (MeDIP-seq)

Genomic DNA was sheared × 3 for 10 min each time using a Bioruptor probe (Diagenode, Belgium) on ice at High setting (30 sec On, 30 sec Off), and passed through Qiagen QIAprep Spin columns. The extent of shearing was confirmed by running 300 ng of each sample on 1.5% agarose gel. All samples were sheared to the same extent, ranging from 100 - 500 bp with the majority of fragments at 200 bp.

Using the Illumina DNA Sample Prep Kit (FC-102-1001-1, Essex, UK), 5 μg of each sheared gDNA sample was end-repaired, adenosine-bases were added to blunt ends and respective adaptors were ligated to DNA fragments, according to manufacturer's instructions. After each step, samples were cleaned using QIAquick Spin columns (Qiagen). Subsequently, samples were heated at 95°C for 10 min and immediately cooled on ice for 10 min. 2.2 μg of single-stranded gDNA was used for MeDIP and the rest stored at -20°C as the input.

MeDIP was performed as previously described [21]. Briefly, this was done using 7.5 μg of 5'methyl-cytosine antibody (MAb-5MECYT-500, Diagenode) in 500 μl IP buffer (10 mM sodium phosphate, pH 7.0, 140 mM NaCl, 0.05% Triton X-100) and incubated for 2.5 h at 4°C whilst rotating. 40 μl of 50% Protein-A agarose slurry (sc-2001, Santa Cruz, Germany) in IP buffer was added and incubated for further 2.5 h, whilst rotating at 4°C. Protein-A agarose beads were subsequently spun down and washed × 3, 10 min each time with IP buffer before eluting with 250 μl of digestion buffer, rotating at 55°C for another 2.5 h. Enriched methylated gDNA was purified using × 2 phenol:chloroform isolation, chloroform wash and precipitation using NaCl. Following washes with 70% ethanol, samples were quantified and a non-saturating amplification was performed using Illumina Primers 1.1 and 1.2 and 14-cycle PCR as recommended by Illumina. Next, samples were cleaned using QIAquick Spin columns and quantified on Bioanalyzer. 20 ng of each sample was used to confirm enrichment of methylated locus (*OXT*) and a concomitant depletion of un-methylated locus (*UBE2B*) versus the input by qPCR, as previously described [21]. MeDIP samples were loaded onto a 2% agarose gel and the 150 - 250 bp bands were cut, and DNA eluted using Qiagen Gel extraction kit and further quantified using Bioanalyzer. Since we used "Illumina Library Single end Primers 1" (92 bp long), we expected our "short libraries" to contain insert sizes to range between 50 - 150 bp long. High throughput sequencing was performed (GeneService, Cambridge, UK) for each of the libraries on 2 channels of the Illumina GAII machine to a sequencing depth of at least 14 mil reads of 35 bp length for each library.

### Data sets, genomic features and data analysis

MeDIP-seq data of human hearts were analyzed using a Bayesian deconvolution strategy, BATMAN (22). BATMAN scores from four normal human hearts were averaged using a Perl script (written by MKC and HGG). MeDIP-seq data of human sperm cells analyzed using the same algorithm came from a published resource [22]. MeDIP-seq data for normal human hearts will be deposited in GEO (Accession number). Average plots of methylation densities were calculated using an algorithm previously described [28]. Transcription factor binding motifs conserved in human/mouse/rat and not containing repetitive elements were from UCSC Genome Browser (http://genome.ucsc.edu/; TFBS Conserved track). ChIP-seq co-ordinates for 17 transcription factors were obtained from ENCODE projects deposited in UCSC Genome Browser and other published work (see references). Intersections between datasets were computed using the Table Browser in UCSC Genome Browser or BEDTools http://sourceforge.net/projects/bedtools/[29].

### CpG island annotation

This was obtained from the UCSC Genome Browser (annotated according to [30]). CpG islands were predicted by searching the sequence one base at a time, scoring each dinucleotide (+17 for CG and -1 for others) and identifying maximally scoring segments. Each segment was then evaluated for the following criteria: GC content of 50% or greater, length greater than 200 bp, ratio greater than 0.6 of observed number of CG dinucleotides to the expected number on the basis of the number of Gs and Cs in the segment.

## Authors' contributions

MKC, HGG, TAD and RSYF carried out the analyses, MM performed the MeDIP experiments, MKC, MRB and RSYF drafted the manuscript. All authors read and approved the final manuscript.
